# Rapid generation of durable B cell memory to SARS-CoV-2 spike and nucleocapsid proteins in COVID-19 and convalescence

**DOI:** 10.1126/sciimmunol.abf8891

**Published:** 2020-12-22

**Authors:** Gemma E. Hartley, Emily S.J. Edwards, Pei M. Aui, Nirupama Varese, Stephanie Stojanovic, James McMahon, Anton Y. Peleg, Irene Boo, Heidi E. Drummer, P. Mark Hogarth, Robyn E. O’Hehir, Menno C. van Zelm

**Affiliations:** 1 Department of Immunology and Pathology, Monash University, Melbourne, VIC, Australia.; 2 Department of Allergy, Immunology & Respiratory Medicine, Central Clinical School, Monash University, Melbourne, VIC, Australia.; 3 Allergy, Asthma and Clinical Immunology, Alfred Health, Melbourne, VIC, Australia.; 4 Department of Infectious Diseases, The Alfred and Central Clinical school, Monash University, Melbourne, VIC, Australia.; 5 Department of Infectious Diseases, Monash Health, Melbourne, VIC, Australia.; 6 Infection and Immunity Program, Monash Biomedicine Discovery Institute, Department of Microbiology, Monash University, Clayton, VIC, Australia.; 7 Viral Entry and Vaccines Group, Burnet Institute, Melbourne, VIC, Australia.; 8 Department of Microbiology and Immunology, Peter Doherty Institute for Infection and Immunity, University of Melbourne, Melbourne, VIC, Australia.; 9 Department of Microbiology, Monash University, Clayton, VIC, Australia.; 10 Immune Therapies Group, Burnet Institute, Melbourne, VIC, Australia.; 11 Department of Pathology, The University of Melbourne, Parkville, VIC, Australia.

## Abstract

Lasting immunity following SARS-CoV-2 infection is questioned because serum antibodies decline in convalescence. However, functional immunity is mediated by long-lived memory T and B (Bmem) cells. Therefore, we generated fluorescently-labeled tetramers of the spike receptor binding domain (RBD) and nucleocapsid protein (NCP) to determine the longevity and immunophenotype of SARS-CoV-2-specific Bmem cells in COVID-19 patients. A total of 36 blood samples were obtained from 25 COVID-19 patients between 4 and 242 days post-symptom onset including 11 paired samples. While serum IgG to RBD and NCP was identified in all patients, antibody levels began declining at 20 days post-symptom onset. RBD- and NCP-specific Bmem cells predominantly expressed IgM^+^ or IgG1^+^ and continued to rise until 150 days. RBD-specific IgG^+^ Bmem were predominantly CD27^+^, and numbers significantly correlated with circulating follicular helper T cell numbers. Thus, the SARS-CoV-2 antibody response contracts in convalescence with persistence of RBD- and NCP-specific Bmem cells. Flow cytometric detection of SARS-CoV-2-specific Bmem cells enables detection of long-term immune memory following infection or vaccination for COVID-19.

## Teaser

Memory B cells specific for SARS-CoV-2 spike and nucleocapsid proteins persist in peripheral blood after recovery from COVID-19.

## INTRODUCTION

Coronavirus disease (COVID)-19 is a global health emergency. The causative agent, severe acute respiratory syndrome coronavirus-2 (SARS-CoV-2) is highly contagious and has infected tens of millions worldwide and caused over 1.2 million deaths since its discovery in Wuhan, China in December 2019 (*1*, *2*). Although mild or asymptomatic in many cases, SARS-CoV-2 infection in the elderly and individuals with chronic health problems can result in severe COVID-19 requiring invasive ventilation or in death (*3*–*6*).

Since early 2020, many insights have been obtained into the pathology of severe COVID-19. It appears that high viral loads induce strong inflammatory responses that cause systemic disease, especially in the elderly and in individuals requiring immunosuppressive treatment (*6*). Immunomodulation with corticosteroids has improved survival in hospitalized individuals, and anti-SARS-CoV-2 monoclonal antibody treatments have shown early evidence of alleviating symptoms and decreasing SARS-CoV-2 viral loads in mild disease (*7*, *8*).

The COVID-19 pandemic has led to a huge global effort to identify a safe therapeutic vaccine to induce a protective immune response. Our current understanding of SARS-CoV-2 immunity is based mainly on previous experiences with SARS-CoV, supplemented with recent studies in patients infected with and recovered from SARS-CoV-2 infection. Similar to SARS-CoV infection (*9*, *10*), the main antibody targets in SARS-CoV-2 are the spike and nucleocapsid proteins (NCP) (*11*–*14*). These antibodies are detectable from approximately 6 days after PCR confirmation of infection, and those directed against spike receptor binding domain (RBD) show neutralizing capacity and hence, can prevent infection (*15*, *16*). However, the rapid decline of anti-SARS-CoV-2 serum IgG levels beyond 20 days post-diagnosis and the transient presence of circulating plasmablasts have led to questions about the longevity of immunity (*17*–*21*). In contrast, antigen-specific memory T cells and memory B (Bmem) cells can be detected in convalescence (*22*–*24*). As these memory cells are programmed to respond rapidly upon subsequent antigen encounter, it is reasonable to hypothesize that these long-lived memory cells provide durable long-term immunity (*4*, *25*). However, detailed insight into the nature and longevity of the Bmem cell compartment specific to SARS-CoV-2 is currently still unresolved (*26*).

We extensively characterized the SARS-CoV-2-specific Bmem cell compartment using unique sets of fluorescently-labeled recombinant tetramers of the SARS-CoV-2 RBD and NCP antigens in combination with an extensive flow cytometry panel. The SARS-CoV-2-specific Bmem cells were quantified and characterized in 36 samples from 25 patients with COVID-19 or in convalescence. Circulating RBD- and NCP-specific Bmem cell subsets were detected early after infection and persisted over 242 days post-symptom onset. Early after infection, antigen-specific Bmem cells predominantly expressed IgM, followed over time by a predominance of IgG1. RBD-specific Bmem cell numbers were found to positively correlate with circulating T_FH_ cell numbers suggesting prolonged germinal center (GC) activity. These analyses highlight that a decline in serum antibodies in convalescence may not reflect waning of immunity, but rather a contraction of the immune response with the development and persistence of B cell memory.

## RESULTS

### Fluorescent NCP and RBD tetramers to identify SARS-CoV-2-specific B cells

**Fig. 1:**
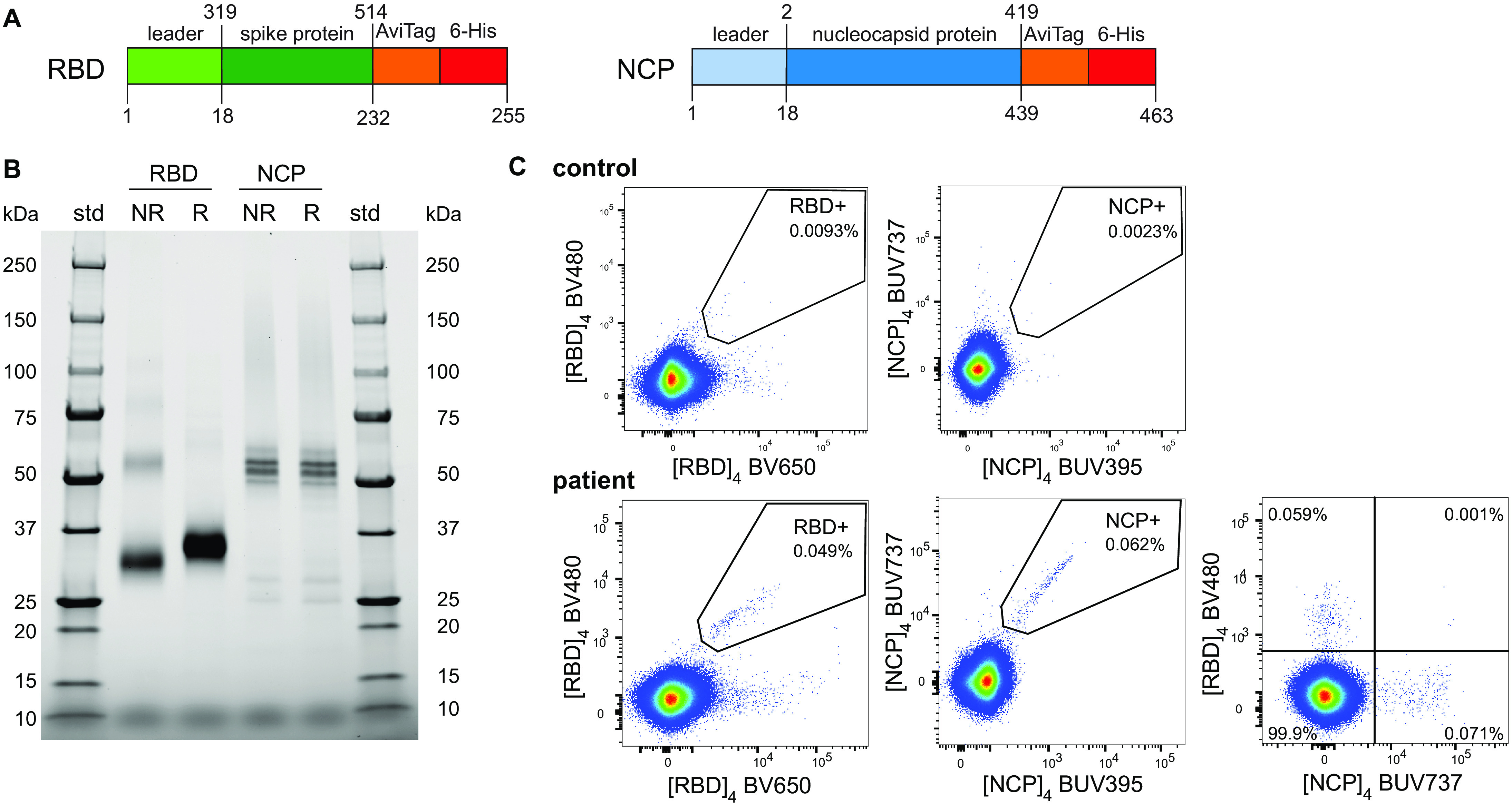
Construct design and detection of SARS-CoV-2 RBD- and NCP-specific B cells (**A**) Recombinant spike receptor binding domain (RBD) and nucleocapsid protein (NCP) constructs of SARS-CoV-2. (**B**) SDS PAGE of purified, reduced (R) or non-reduced (NR) recombinant RBD and NCP. (**C**) Flow cytometry stainings of CD19^+^ B cells from an uninfected control and a recovered COVID-19 patient using double discrimination through inclusion of two fluorescent tetramers for each protein (RBD or NCP) in the same staining tube. Percentages indicate the proportions of RBD- or NCP-specific cells within total CD19^+^ B cells.

The antigen-specific B cell response to SARS-CoV-2 was characterized using recombinant forms of the RBD and NCP. Both proteins were generated in Expi293F cells with an AviTag for targeted biotinylation and tetramerization with fluorescently-labeled streptavidins to minimize epitope masking (Fig. 1, A and B). Two tetramers with distinct fluorochromes were generated for each protein: BV480 and BV650 for RBD, and BUV395 and BUV737 for NCP. In subjects with a history of COVID-19, distinct populations of RBD-specific and NCP-specific B cells were detected using double-discrimination (Fig. 1, C). Detection of these populations was highly specific, because neither population was detected in non-infected controls, and the RBD- and NCP-tetramers stained distinct B cell subsets (Fig. 1, C).

### COVID-19 patient clinical and immunological characteristics

The SARS-CoV-2-specific antibody and B cell response was investigated in 25 COVID-19 confirmed patients with samples obtained between 4 and 242 days after symptom onset (table S1). Patients were classified into three levels of disease severity (*27*): six with severe disease requiring respiratory support in the intensive care unit (ICU); three with moderate disease requiring non-ICU hospital admission, and 16 with mild disease managed in the community (table S1). Eleven patients were sampled twice (paired samples); first between 21-106 days post-symptom onset and again at 116-242 days to evaluate the longevity of the Bmem cell response to SARS-CoV-2 infection (table S1). At the time of blood sampling, the majority of patients had normal blood counts of the major leukocyte and lymphocyte subsets (table S2). Of note, the three patients sampled within the first 14 days post-symptom onset showed CD3^+^ T-cell lymphopenia due to reduced CD8^+^ T-cell counts (table S2). All patients exhibited normal absolute numbers of B cells (table S2).

### COVID-19 patients generate neutralizing, RBD- and NCP-specific antibodies

**Fig. 2:**
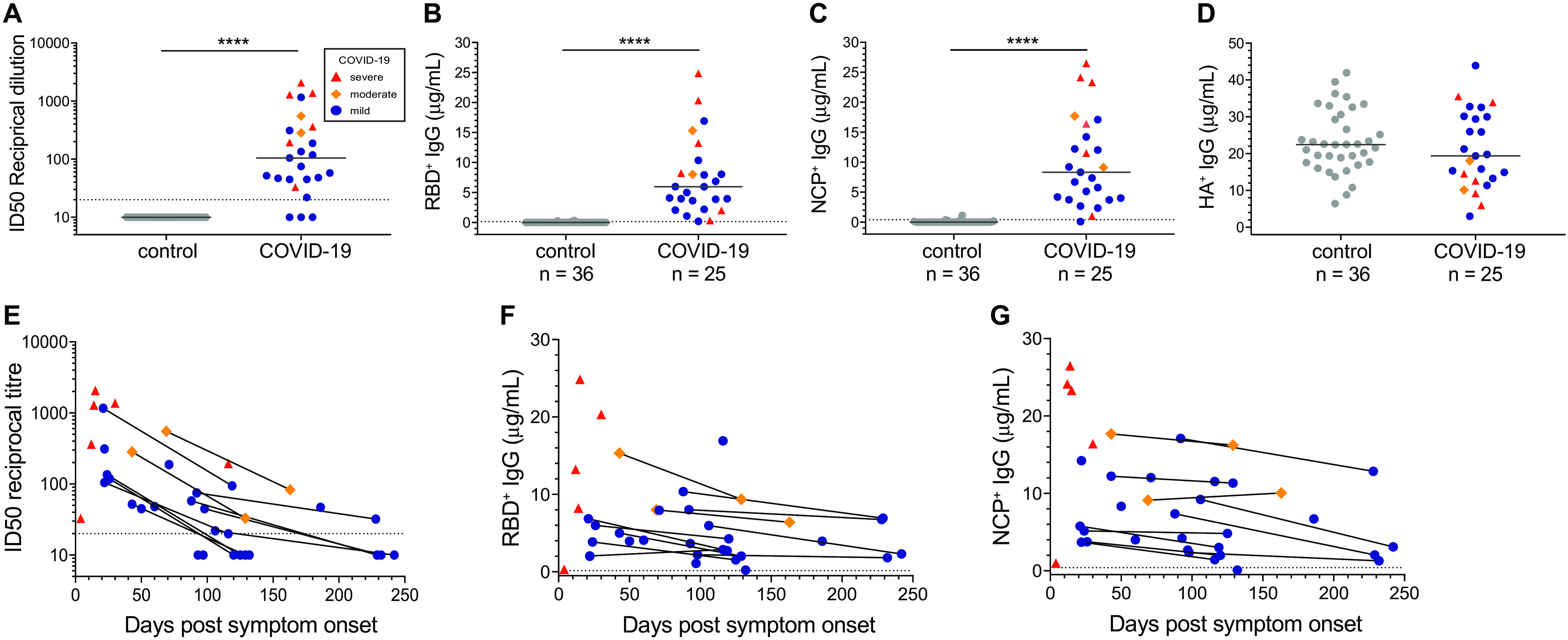
Neutralizing antibodies and RBD-, and NCP- and HA-specific IgG antibody levels. (**A**) Neutralizing antibody titers to SARS-CoV-2 in 25 COVID-19 patients and 36 historic controls (sampled in 2019 and Q1 2020) as determined using a pseudovirus assay. Antigen-specific plasma IgG levels were determined to (**B**) SARS-CoV-2 RBD, (**C**) SARS-CoV-2 NCP and (**D**) influenza A/Michigan/2015 haemagglutinin (HA). Horizontal solid gray lines represent median values. (**E**) Neutralizing antibody titers, and IgG levels to (**F**) RBD and (**G**) NCP plotted against time since symptom onset of infection of 25 patients including 11 patients sampled twice. Patient datapoints are marked based on disease severity with severe as red triangles, moderate as orange diamonds and mild as blue circles. The 11 paired samples are connected with black lines. The dotted horizontal lines in panels A and E depict an ID50 of 20, the cut-off for neutralization (*68*). The dotted horizontal lines in B, C, F and G depict the cut-off for positivity, defined as +2SD of the controls. Statistics were performed with the Mann-Whitney *U-*test for unpaired data; **** *p* ≤ 0.0001.

The humoral response to SARS-CoV-2 infection in all patients was examined with a pseudovirus neutralization assay. Neutralizing antibody titers to SARS-CoV-2 were detected in 22/25 patients, whereas none of the 36 uninfected controls had detectable neutralizing titers (Fig. 2, A). IgG ELISAs were performed to both SARS-CoV-2 RBD and NCP proteins in 25 patients and 36 controls. All 25 patients were positive for RBD-specific IgG and 24/25 were positive for NCP-specific IgG, i.e., 2 standard deviations (2 SD) above the median of healthy controls (Fig. 2, B and C). All controls and patients had detectable levels of IgG to hemagglutinin (HA) of influenza H1N1 strain A/Michigan/45/2015 (Fig. 2, D) (*28*), which was a recommended strain in the quadrivalent annual vaccine from 2017-2019 (*29*). There was no significant difference in HA antibody levels between the patient and control groups. Thus, the recombinant SARS-CoV-2 RBD and NCP proteins are recognized by antibodies in COVID-19 patients with high sensitivity and specificity.

The neutralization titers (ID50), and RBD- and NCP-specific IgG levels in our patients declined over time in convalescence (Fig. 2, E-G). Neutralizing antibody titers were highest in patients sampled approximately 20 days post-symptom onset and subsequently contracted (Fig. 2, E). All ID50 titers were lower in the second sample of the 11 paired samples, and 7/11 repeat samples were at or below the threshold of neutralizing capacity (ID50 of 20) (Fig. 2, E). In parallel, RBD- and NCP-specific IgG levels were highest in the patients sampled around 20 days post-symptom onset, and in 10/11 repeat samples the RBD- and NCP-specific IgG levels were lower than the first draw (Fig. 2, F and G). Still, the decline after 20 days seemed to reach a plateau between 120-240 days with nearly all samples having detectable levels of RBD- and NCP-specific IgG.

### Detailed immune profiling of SARS-CoV-2-specific memory B cells

**Fig. 3:**
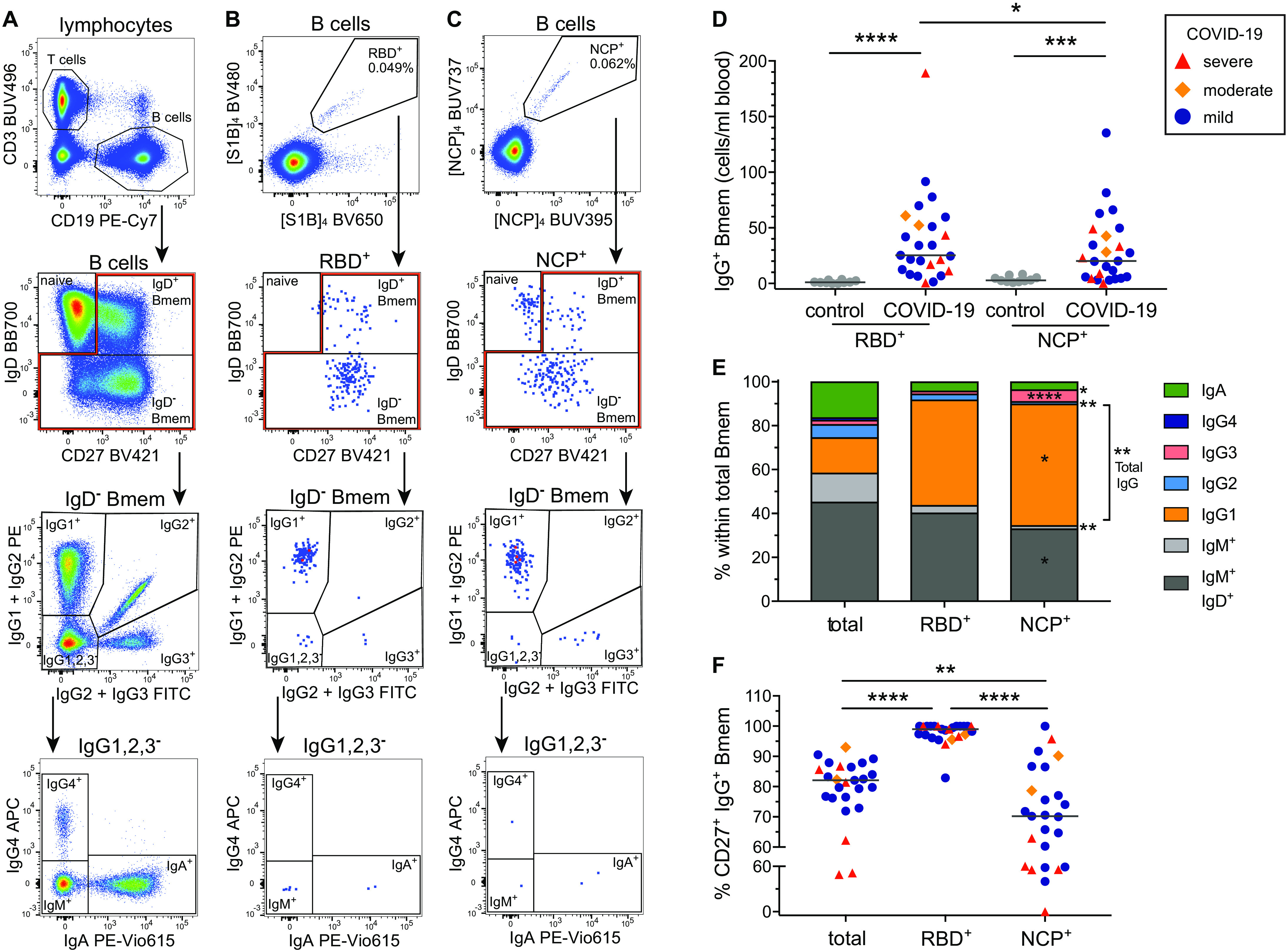
RBD- and NCP-specific Bmem cells predominantly express IgM or IgG1. (**A**) Gating strategy to discriminate T and B cells, followed by subsetting of total B cells into CD27^-^IgD^+^ naive, CD27^+^IgD^+^ Bmem and CD27^+/−^IgD^-^ Bmem cells. Within IgD^-^ Bmem cells, Ig switched subsets were defined based on the differential expression of IgG1, 2, 3, 4 subclasses and IgA. (**B**) Detection of RBD-specific (RBD^+^) B cells, and (**C**) NCP-specific (NCP^+^) B cells, utilized the same gating strategy as for total B cells. (**D**) Absolute numbers of IgG^+^ RBD^+^ and NCP^+^ Bmem cells in the first sample of 25 COVID-19 patients and 10 uninfected healthy controls. (**E**) Median frequencies of total, RBD^+^ and NCP^+^ Bmem subsets in 25 COVID-19 patients. Significant differences between RBD^+^ and NCP^+^ Bmem subsets are depicted with asterisks in the NCP column. (**F**) Frequencies of IgG^+^ Bmem cells expressing CD27 within total, RBD^+^ and NCP^+^ Bmem cells. Patient datapoints are marked based on disease severity with severe as red triangles, moderate as orange diamonds and mild as blue circles. Statistics: panel D, Mann-Whitney *U-*test for unpaired data; panels E and F, Wilcoxon matched-pairs signed rank test for paired samples; * *p* < 0.05, ** *p* < 0.01, *** *p* < 0.001, **** *p* < 0.0001.

To examine the nature and kinetics of the RBD- and NCP-specific Bmem following SARS-CoV-2 infection, the RBD and NCP proteins were biotinylated and tetramerized with fluorescently-labeled streptavidins. RBD- and NCP-specific B cells were evaluated by flow cytometry in all 36 samples for expression of markers for plasmablasts (CD38), activated (CD71) and resting (CD27) Bmem cells, as well as surface IgD, IgA and IgG1, 2, 3 and 4 subclasses (Fig. 3, A) (Table S3). Patients 1-3, sampled between 5-14 days post-onset of symptoms showed a large population of CD38^high^ CD27^+^ plasmablasts, whereas this population was negligible in any of the samples taken >20 days post-onset of symptoms (fig. S1). Bmem cells were defined using IgD and CD27 (Fig. 3, A-C). All patients had detectable numbers of both IgG^+^ RBD- and NCP-specific Bmem cells, which were significantly higher than those of uninfected controls (*p* <0.0001 and *p* = 0.0005 respectively) (Fig. 3, D). The RBD- and NCP-specific Bmem cell populations contained both unswitched (CD27^+^IgM^+^IgD^+^) and immunoglobulin (Ig) class-switched cells (CD27^+/−^IgD^-^) (Fig. 3, B and C). The latter subset predominantly contained IgG1-expressing Bmem cells with smaller proportions expressing IgG3 or IgA (Fig. 3, E). These distributions differed significantly between RBD- and NCP-specific Bmem cells: RBD-specific Bmem cells comprised significantly larger proportions of IgM^+^ IgD^+^, IgM only, IgG2 and total IgG expressing Bmem cell subsets than NCP-specific Bmem cells (Fig. 3, E). Compared to NCP-specific IgG^+^ Bmem cells, a higher proportion of RBD-specific IgG^+^ Bmem cells expressed CD27, a marker associated with increased replication and somatic hypermutation levels in Ig genes (Fig. 3, F) (*30*). Irrespective of the specificity, the proportions of IgG^+^ Bmem expressing CD27 were lower in patients sampled within 25 days post-symptom onset (fig. S2). Thus, SARS-CoV-2 infection induces robust Bmem cell responses, which are predominantly comprised of IgM^+^ and IgG1^+^ Bmem cells with distinct immunophenotypes for those directed against RBD versus NCP.

### Long-term persistence of RBD- and NCP-specific Bmem expressing IgG

**Fig. 4:**
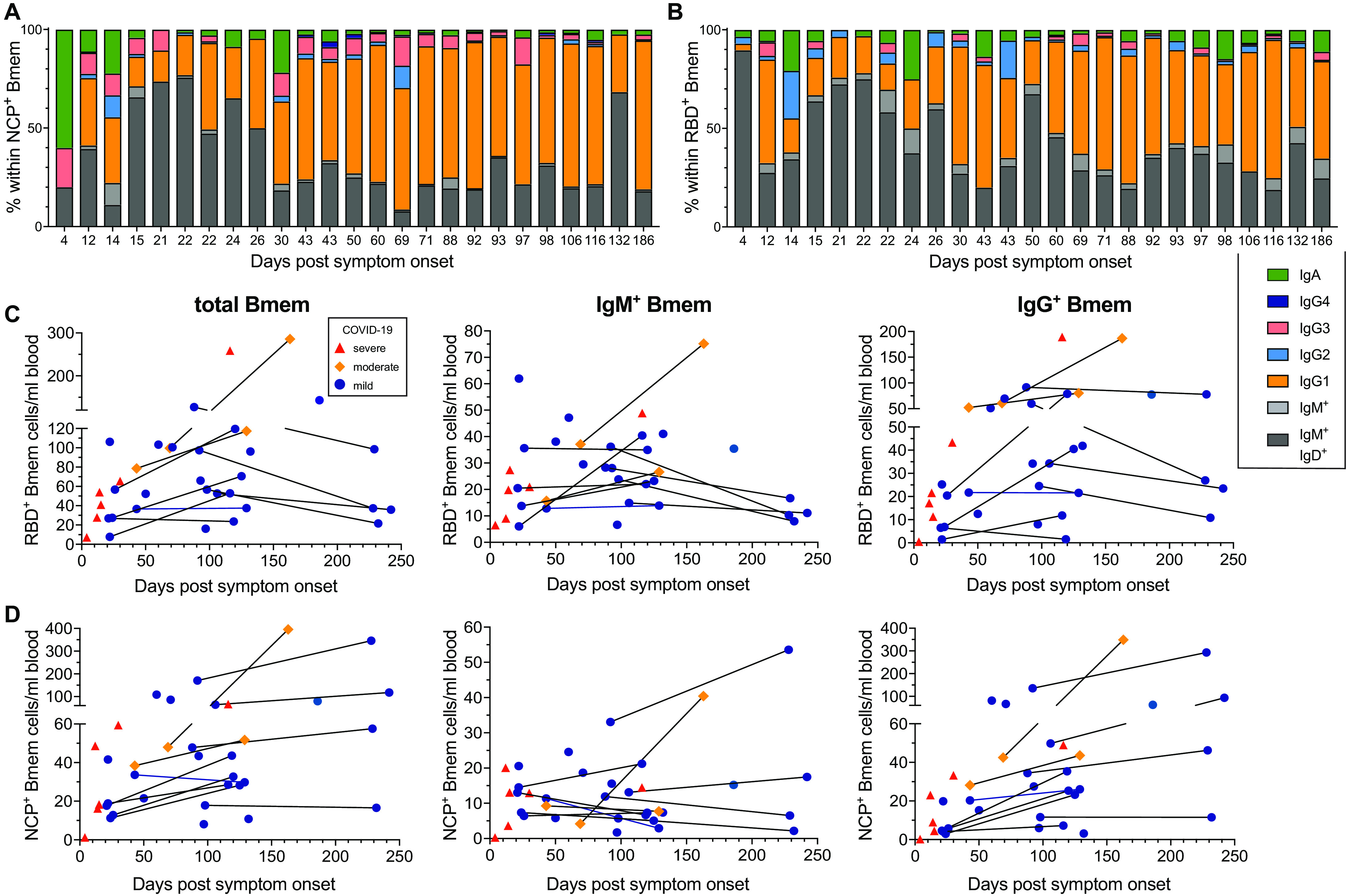
Composition and kinetics of SARS-CoV-2 RBD- and NCP-specific Bmem in convalescence. Relative composition of the Bmem cell compartment based on Ig isotype and IgG subclass expression within (**A**) RBD-specific (RBD^+^) and (**B**) NCP-specific (NCP^+^) Bmem subsets. Patients’ data are ordered by days post-symptom onset. Absolute numbers of total, IgM^+^ and IgG^+^ Bmem cells specific for (**C**) RBD^+^ or (**D**) NCP^+^. Samples are plotted by days post-symptom onset for 25 individuals, with 11 patients sampled twice and paired samples connected with gray lines. Patient datapoints are marked based on disease severity with severe as red triangles, moderate as orange diamonds and mild as blue circles.

The numbers and Ig isotype distribution of RBD- and NCP-specific Bmem cell subsets varied between individuals. However, similar trends were still observed for both subsets with higher proportions and absolute numbers of IgG1^+^ RBD- and NCP-specific Bmem cells in samples taken 26 days or more post-symptom onset (Fig. 4, A and B, fig. S3). RBD-specific Bmem cell numbers were highest between 100-150 days post-symptom onset (Fig. 4, C). Total and IgM^+^ Bmem cells in paired samples taken >200 days were lower than in the corresponding first samples, whereas IgG^+^ Bmem cells remained stable. NCP-specific Bmem cell numbers increased over the first 150 days as well, and in contrast to RBD-specific Bmem cells, they did not decline between 150-240 days (Fig. 4, D). RBD- and NCP-specific Bmem cells trended to be lower in patients with severe COVID-19 (Fig. 3D), although this might be related to the fact that most patients were sampled at earlier timepoints (<30 days) than those with mild or moderate disease (Fig. 4C, D). IgM^+^ and IgG^+^ Bmem cells are predominantly generated in GC responses with T-cell help (*30*). While total, CD4^+^, CD8^+^ and γδ^+^ T-cell numbers did not change over time beyond 26 days post-symptom onset, the CD4^+^ T_reg_, CD4^+^ T_FH_, CD4^+^ T_FR_ and the CD8^+^ T_FH_ subsets all trended to increase over the first 150 days followed by a plateau until 240 days (fig. S4). Of these subsets, the CD4^+^ T_FH_, CD4^+^ T_FR_ and CD8^+^ T_FH_ cell numbers showed a significant positive correlation with RBD-specific total, IgM^+^ and IgG^+^ Bmem cells across the 36 samples (fig. S5). In contrast, only CD8^+^ T_FH_ cell numbers showed a significant correlation with NCP-specific total and IgG^+^ Bmem cells (fig. S6).

In summary, RBD- and NCP-specific IgG and Bmem cells were detected in all 25 patients with a history of COVID-19. While specific IgG levels in serum declined with time post-symptom onset, SARS-CoV-2-specific Bmem cell numbers persisted, and RBD-specific Bmem cell numbers correlated with T_FH_ cell numbers. This long-term presence of circulating Bmem cell subsets directed against both the major SARS-CoV-2 neutralization target (RBD) and a non-neutralizing target (NCP) is indicative of persisting immune memory following natural exposure and could provide a means to evaluate protection from severe COVID-19 following vaccination.

## DISCUSSION

We have shown that COVID-19 patients rapidly generate B cell memory to both the spike and nucleocapsid antigens following SARS-CoV-2 infection. Although rare events, numbers of Bmem cells specific for the major neutralization target, RBD, and a major non-neutralizing target, NCP, were both in the same order of magnitude. Using an extensive flow cytometry panel, we show that in line with typical T-cell dependent responses, IgM^+^ Bmem cells predominated in the first 20 days post-infection, followed by a gradual increase in IgG1^+^ Bmem cells. The fact that nearly all RBD-specific IgG^+^ Bmem cells expressed CD27 and their numbers correlated with circulating T_FH_ cells is indicative of long-lived immune memory.

None of our patients showed a reduction in total B cell numbers, even those sampled within 14 days of symptom onset. Several studies have reported a decrease in B cell frequencies during COVID-19 infection which normalized in convalescence (*31*–*34*). As these studies report frequencies rather than absolute numbers, the reported decrease might have been confounded by an increase in other immune subsets such as NK cells (*31*, *33*).

Patients sampled within 14 days of symptom onset had an enlarged plasmablast population that was absent in those sampled beyond 20 days post-symptom onset and who are in convalescence. Others have identified this transient plasmablast expansion in COVID-19 (*19*, *31*). However, this observation is not restricted to SARS-CoV-2 infection and has been reported in other viral infections including influenza and dengue virus (*35*, *36*). Quantification of total B cell subsets is important for the monitoring of disease progression and reconstitution of the humoral response post-infection. However, while alterations seen in SARS-CoV-2 infection at this level can be indicative of severity of infection or clinical disease, they are not unique and hence not indicative of long-lived SARS-CoV-2-specific humoral responses.

The detection of virus-specific antibodies is regularly employed in the diagnosis of other viral infections including Epstein-Barr virus, cytomegalovirus, hepatitis B virus, and influenza virus (*37*–*40*). The antibody response to SARS-CoV-2 infection has been shown to be directed to multiple targets of the virus including the spike protein, with those that target the RBD considered neutralizing (*11*, *20*, *21*, *26*). Other antibodies target the NCP (*11*, *20*, *21*) or non-structural proteins (*41*). Detection of such antibodies can be used as markers of recent infection. However, it has been reported that antibody levels to SARS-CoV-2 decrease over time (*20*, *21*, *42*). What we now show is that this decrease reflects a contraction of the immune response. Despite the decline in antibody levels, detectable levels of antibodies remain until 240 days post-symptom onset and their presence is accompanied by the persistence of RBD- and NCP-specific Bmem cells. These antigen-specific Bmem are rapidly generated and particularly those expressing IgG^+^ remain numerically high, and hence may represent a more robust marker of long-term immune memory.

Antigen-specific Bmem cells are very rare events at 0.008-0.1% of B cells. Here, we used double-discrimination to exclude B cells that non-specifically bound to the fluorochrome, which is typically observed when utilizing large protein-based fluorochromes (e.g., phycoerythrin (PE) and allophycocyanin (APC)) (*22*, *33*, *34*, *43*–*46*). To further overcome this limitation, we used non-protein polymer fluorochromes, which exhibited minimal non-specific B cell binding and increased the sensitivity of our assay (*28*).

Our study shows the kinetics and longevity of SARS-CoV-2-specific Bmem cell numbers. Other studies have identified SARS-CoV-2-specific B cells in COVID-19 patients with particular focus on the RBD of the spike protein, mainly for the purpose of cloning neutralizing SARS-CoV-2-specific antibodies (*4*, *14*, *22*, *47*, *48*). Such studies have observed a predominant IgG^+^ B cell response to SARS-CoV-2 with lower frequencies of cells expressing IgM and IgA (*22*, *45*, *46*). We have expanded on this through detailed flow cytometry with the inclusion of absolute cell counts to show that SARS-CoV-2-specific Bmem cells predominantly expressed IgM or IgG1. This distinction enabled the discrimination between the initially large fraction of IgM^+^ Bmem cells that tended to decline beyond 150 days and the IgG-expressing fraction persisted. These IgM^+^ and IgG^+^ Bmem waves appear to be reproducible and have also been observed in another recent study (*49*).

The Bmem cell populations directed against the two SARS-CoV-2 targets showed remarkable differences. The RBD-specific Bmem cells were nearly all CD27^+^ and strongly correlated with T_FH_ cells, while this was not observed for NCP-specific Bmem cells. Differential expression of CD27 and IgG subclasses on human Bmem cells are associated with different maturation stages (*30*, *50*). Typically, CD27^+^ Bmem cells have more somatic hypermutations and have undergone more cell divisions than those lacking CD27 expression (*30*, *50*). Limited GC activity has been reported in COVID-19 patients early in convalescence (15-36 days post-infection) (*34*). It would be of interest to study whether GC activity increases or persists beyond 36 days. Similarly, detailed molecular studies and somatic hypermutation analysis of RBD-specific Bmem could provide insights into prolonged GC activity.

We here show long-term persistence of SARS-CoV-2-specific Bmem cells with kinetics that suggest high durability, particularly of those expressing IgG. Bmem cells show evidence of antigen experience through extensive replication cycles, elevated somatic hypermutation levels and class switch recombination (*30*, *50*). After infection, a portion of Bmem cells can be detected with an ‘activated’ phenotype (CD21^lo^ CD27^+^) and this population has been shown to contract after ~2 weeks (*51*, *52*). The remaining Bmem cells are defined as ‘resting’ with a predominant CD21^+^ CD27^+^ phenotype. Furthermore, Bmem cells have been shown to increase expression of surface molecules including CD80, CD180 and TACI indicating the potential for rapid activation upon antigen re-encounter (*30*, *53*). While these markers have not been assessed in SARS-CoV-2-specific Bmem cells, they display the classical surface markers (i.e., CD27^+/−^ IgG^+^) indicating long-lived memory. There have been some studies reporting that the Bmem cell response to SARS-CoV may not be long-lived (*54*, *55*), however, our results indicate that SARS-CoV-2 infection generates long-lasting B cell memory up to 8 months post-infection that could be protective against systemic disease upon reinfection.

In this study, we sampled peripheral blood and hence measured the systemic Bmem cell response to SARS-CoV-2 infection. We know from vaccination studies in mice and humans that local and systemic Bmem cells are phenotypically different (*56*, *57*). It has also been shown that influenza-specific Bmem cells persist in the lungs of mice and do play a role in protection upon reinfection (*58*, *59*). However, at present the attributes of SARS-CoV-2 immunity in the respiratory tract are largely unknown. As knowledge of SARS-CoV-2 and human lung immunology evolve, we will gain insight into what is required for a protective response to this respiratory virus. However, we propose that the establishment of systemic immune memory will prevent severe systemic COVID-19, and reinfection may be limited to a mild or asymptomatic upper respiratory tract infection.

The identification and analysis of SARS-CoV-2-specific Bmem cells could potentially be used as a surrogate marker of humoral immunity in vaccination studies. Currently, SARS-CoV-2 vaccination trials focus predominantly on SARS-CoV-2-specific and neutralizing antibodies as markers of vaccine efficacy (*5*, *60*–*64*). Serum antibody levels decline following antigen clearance as part of the contracting immune response. As we have shown that SARS-CoV-2-specific Bmem cell numbers are stable over time, we propose that these Bmem may represent a more robust marker of long-lived humoral immune responses than serum antibodies. Therefore, cellular measurements of the immune response could be more reliable markers for maintenance of immunity following natural infection or vaccination.

## METHODS

### Participants

Individuals with a PCR-confirmed diagnosis of COVID-19 and uninfected controls were enrolled in research studies to examine their peripheral blood B- and T-cell subsets (projects: Alfred Health Human Research and Ethics Committee Numbers 182/20 and 202/20, Monash University 2016-0289 and 2020-26385). From March to September 2020, 25 individuals with a history of PCR-confirmed COVID-19 disease and 36 healthy controls (sampled in 2019 and Q1 2020) consented to one or two 40 ml donations of blood as well as the collection of clinical data including: basic demographics (age, sex), clinical details of COVID-19 (clinical symptoms, date of symptom onset, and COVID-19 specific treatments) and co-morbid medical conditions (table S1). This study was conducted according to the principles of the Declaration of Helsinki and approved by local human research ethics committees.

### Sample processing

Blood samples of patients and controls were processed as described previously (*28*, *65*). Briefly, 200 μl was used for whole blood cell counts (Cell Dyn analyzer; Abbott core laboratory, Abbott Park, IL) and Trucount analysis (see flow cytometry section). The remainder was used to separate and store plasma (-80°C), and to isolate live peripheral blood mononuclear cells (PBMC) by Ficoll-Paque density gradient centrifugation and cryopreservation at a cell density of 10 million cells/ml in RPMI medium with 40% FCS and 10% DMSO in liquid nitrogen for later analysis of SARS-CoV-2-specific B cells.

### Protein production and tetramerization

Recombinant nucleocapsid protein (NCP) and receptor binding domain of the spike protein (RBD) of SARS-CoV-2 (GenBank: MN908947.3) were produced with a human Ig leader and the Fel d 1 leader sequence, respectively. Each protein construct was C-terminally fused to the biotin ligase (BirA) AviTag target sequence and a 6His affinity tag (Fig. 1, A). The DNA constructs were cloned into a pCR3 plasmid and produced and purified as described previously (*28*). Briefly, plasmid DNA was purified from *E. coli* by Maxiprep (Zymo Research, Irvine, CA), and 30 μg DNA was transfected into 293F cells using the Expi293 Expression system (Thermo Fisher, Waltham, MA). Supernatants from 25 ml cultures were collected on days 3 (NCP) or 5 (RBD) post-transfection and purified by application to a Talon NTA-cobalt affinity column (Takara Bio, Kusatsu, Shiga, Japan) with elution in 200 mM imidazole. Eluted proteins were then dialyzed against 10 mM Tris for 48 hours at 4°C. Purified proteins were biotinylated by incubating at room temperature overnight with 1/8 of final volume each of Biomix A (0.5 M Bicine-HCl, pH8.3) and Biomix B (100 mM ATP, 100 mM MgOAc, 500 μM D-biotin) followed by 2.5 μg of BirA enzyme per milligram of protein. Biotinylated protein was subsequently dialyzed against 10 mM Tris for 36 hours at 4°C, and subsequently stored at -80°C prior to use. Soluble biotinylated NCP protein was tetramerized by the addition of either Brilliant Ultra Violet (BUV)395-conjugated streptavidin, or streptavidin-BUV737, and biotinylated RBD with streptavidin-BV480 or streptavidin-BV650 (BD Biosciences, Franklin Lakes, NJ) at a protein:streptavidin molar ratio of 4:1 making 4 unique tetramers: [NCP]_4_-BUV395, [NCP]_4_-BUV737, [RBD]_4_-BV480 and [RBD]_4_-BV650.

### SDS-PAGE

SDS-PAGE analyses were performed as described previously (*28*)*.* Briefly, 10 μl of sample was mixed with 2.5 μl of 4X Laemmli Sample buffer (non-reducing) (BioRad, Hercules, CA) or reducing buffer (4X Laemmli Sample buffer with the addition of 1.25 μl DTT). Samples under reducing conditions were heated to 85°C for 10 min. 10 μl of ladder (1:1 mixture) of Precision plus protein standard (Unstained and All blue, both from BioRad), and reduced or non-reduced sample was loaded on a 4-15% Mini-PROTEAN TGX Stain-Free gel (BioRad) and run for 30 min at 200 V then imaged on the BioRad ChemiDoc Touch imaging system (BioRad).

### Measurement of SARS-CoV-2 neutralizing antibodies in plasma

Measurement of neutralizing antibodies was performed using SARS-CoV-2 retroviral pseudotyped particles and a 293T-ACE2 cell line (*66*) as described before (*67*). Plasma was heat inactivated at 56°C for 45 min followed by serial dilution in DMF10. Each dilution was mixed in duplicate with an equal volume of SARS-CoV-2 (WUHAN-1 spike) retroviral pseudotyped virus and incubated for 1 hour at 37°C. Virus-plasma mixtures were added to 293T-ACE2 cell monolayers in 96-well poly-L-lysine coated plates seeded the day prior at 10,000 cells/well, and incubated for 2 hours at 37°C before addition of an equal volume of DMF10 and incubated. After 3 days, tissue culture fluid was removed, monolayers were washed once with PBS and lysed with cell culture lysis reagent (Promega, Madison, WI) and luciferase measured using luciferase substrate (Promega) in a Clariostar plate reader (BMG LabTechnologies, Offenburg, Germany). The mean percentage entry was calculated as (relative light units (RLU) plasma+virus)/(RLU medium+virus)*100. The percentage entry was plotted against the reciprocal dilution of plasma in GraphPad Prism 8 Software (GraphPad Software, La Jolla, CA) and curves fitted with a one-site specific binding Hill plot. The reciprocal dilution of plasma required to prevent 50% virus entry was calculated from the non-linear regression line (ID50). The lowest amount of neutralizing antibody detectable is a titer of 20 (*68*). All samples that did not reach 50% neutralization were assigned an arbitrary value of 10.

### ELISA

EIA/RIA plates (Costar, St Louis, MO) were coated with 2 μg/ml recombinant SARS-CoV-2 NCP or RBD or with hemagglutinin (HA) from influenza A/Michigan/08/2015 (AM15) overnight at 4°C (*28*). Plates were subsequently blocked with 3% BSA in PBS and incubated with diluted plasma samples, 1:30 for RBD and NCP and 1:50 for AM15. Antigen-specific IgG was detected by adding rabbit anti-human IgG HRP (Dako, Glostrup, Denmark). ELISA plates were developed using TMB solution (Life Technologies, Carlsbad, CA) and the reaction was stopped with 1 M HCl. Absorbance (OD450nm) was measured using a Multiskan Microplate Spectrophotometer (Thermo Fisher). Serial dilutions of recombinant human IgG (in-house made human Rituximab) in separate wells on the same plate were performed for quantification of specific IgG.

### Flow cytometry

Absolute numbers of leukocyte subsets were determined as described previously (*28*, *65*). Briefly, 50 μl of whole blood was added to a Trucount tube (BD Biosciences) together with a 20 μl antibody cocktail containing antibodies to CD3, CD4, CD8, CD16, CD19, CD56 and CD45 (tables S3 and S4) and incubated for 15 min at room temperature in the dark. Subsequently samples were incubated for a further 15 min at room temperature with 500 μl of 0.155 M NH_4_Cl to lyse red blood cells. The tube was then stored in the dark at 4°C for up to 2 hours prior to acquisition on the LSRII analyzer (BD Biosciences).

Detailed T-cell subsetting was performed with two 11-color flow cytometry panels (table S3) as previously described (*65*). Specific subsets were defined as follows: CD4^+^ T_reg_, CD3^+^CD4^+^CD8^-^CD127^-^CD25^+^; CD4^+^ T_FH_, CD3^+^CD4^+^CD8^-^CD45RA^-^CXCR5^+^; CD4^+^ T_FR_, CD3^+^CD4^+^CD8^-^CD127^-^CD25^+^CD45RA^-^CXCR5^+^; CD8^+^ T_FH_, CD3^+^CD4^-^CD8^+^CD45RA^-^CXCR5^+^ (fig. S4).

For detection of antigen-specific B cells, 12.5 million PBMC were incubated with fixable viability stain 700 (BD Biosciences), antibodies against CD3, CD19, CD21, CD27, CD38, CD71, IgA, IgD, IgG1, IgG2, IgG3, IgG4, (tables S3 and S4) and 5 μg/ml (total of 1.25 μg per 250 μl stain) each of [NCP]_4_-BUV395, [NCP]_4_-BUV737, [RBD]_4_-BV480 and [RBD]_4_-BV650 for 15 min at room temperature in a total volume of 250 μl FACS buffer (0.1% sodium azide, 0.2% BSA in PBS). In addition, 5 million PBMC were similarly incubated with fixable viability stain 700 (BD Biosciences), antibodies against CD3, CD19, CD27 and IgD, plus BUV395-, BUV737-, BV480- and BV650-conjugated streptavidin controls (tables S3 and S4). Following staining, cells were washed twice with FACS buffer and filtered through a 70 μM filter prior to acquisition on the 5-laser BD LSRFortessa X-20. Flow cytometer set-up and calibration was performed using standardized EuroFlow SOPs, as previously described (tables S3 and S4) (*28*, *65*, *69*).

### Data analysis and statistics

All flow cytometry data were analyzed with FlowJo v10 software (TreeStar, Ashland, Ore). Statistical analysis was performed with GraphPad Prism 8 Software (GraphPad Software). Matched pairs were analyzed with the non-parametric Wilcoxon matched pairs signed rank test. Unpaired groups were analyzed with the non-parametric Mann-Whitney *U*-test. Correlations were performed using the non-parametric Spearman’s rank correlation. For all tests, *p* < 0.05 was considered significant.

## SUPPLEMENTARY MATERIALS

immunology.sciencemag.org/cgi/content/full/5/54/eabf8891/DC1 Figure S1. Absolute numbers of plasmablasts in COVID-19 patients Figure S2. Frequencies of RBD- and NCP-specific IgG^+^ Bmem cells expressing CD27 Figure S3. Absolute numbers and frequencies of RBD- and NCP-specific Bmem cells expressing distinct Ig isotypes and IgG subclasses Figure S4. Absolute numbers of T helper cell subsets in COVID-19 patients Figure S5. Correlations between absolute numbers of RBD-specific Bmem and T helper cell subsets Figure S6. Correlations between absolute numbers of NCP-specific Bmem and T helper cell subsets Table S1. Patient characteristics Table S2. Immunological details of patients Table S3. Composition of the antibody panels Table S4. Antibody list Table S5. Flow cytometer set-up Table S6. Target values for 7^th^ peak of rainbow beads in fluorescent channels Table S7. Raw data file (Excel spreadsheet)
